# Ethical implications in using robots among older adults living with dementia

**DOI:** 10.3389/fpsyt.2024.1436273

**Published:** 2024-09-25

**Authors:** Blanca Deusdad

**Affiliations:** Medical Anthropology Research Centre, Department Anthropology, Philosophy and Social Work, Rovira i Virgili University, Tarragona, Spain

**Keywords:** robots, older adults, dementia, ethics, formal caregivers, informal caregivers, co-creation, welfare technology

## Abstract

The aging of the world’s population due to accelerating demographic shift on all continents is causing increasing pressure worldwide, giving rise to a “crisis of care” or “care wave.” The increase in longevity is resulting in an increase in chronic diseases (such as dementia), an increase in care needs to perform the activities of daily living, and situations of isolation and profound loneliness among older adults. These circumstances are opening the debate on the need to use technology, such as robots, to improve the wellbeing of older adults and their caregivers. The aim of this paper is to address the ethical questions in using social and companion robots for people with dementia, such as concerning consent, the replacement of human care, the potential for increased dependency, and the burden on caregivers. Involving older adults and other stakeholders offers the potential to pursue robotics to support older people while also ensuring a strong ethical commitment. The study is a review of high-impact articles on the topic of the use of social and companion robots with older people with dementia.

## 1 Introduction

The concept of “social robots” emerged within academic discourse during the early 21st century, with scholars primarily defining them based on their form, functions, and technical autonomy (1). These robots are characterized by their ability to interact with humans and generate expected behaviors during engagement (2–4).”Companion robots,” on the other hand, refer to “pet-type robots” that accompany patients, particularly those living with dementia, alleviating their discomfort, improving mood, and mitigating loneliness and isolation (5, 6). However, there is no unified use of the terms. While some researchers emphasize emotional relationships and attachment, focusing on robots like PARO (7), others include cognitive tools with emotional and social focuses, such as MARIO (6). Throughout the article, I refer to both social and companion robots, designating social robots as those focusing on cognitive activities and companion robots as those focusing on emotional and attachment relationships. Both types of robots have demonstrated positive impacts on loneliness and isolation.

However, the debate is open regarding the ethical implications of their use in caring for vulnerable populations such as older people with dementia. In general, there is no clear position but opinions that fluctuate from ‘gerotechnological optimism’ (8, 9), which sees the utility of robots in avoiding isolation and loneliness, to warnings of the risk of greater segregation and exclusion of older adults and the replacement of human care by machines (10, 11). From an engineering and psychological perspective some aspects have already been identified. However, further research from an anthropological and sociological standpoint is still needed to better understand the ethical implications and the long-term perspective of older adults and their informal caregivers while taking into account the social and cultural implications and how these interact within the groups. The aim of this paper is to contribute to the debate on the use of social and companion robots for caring for older people with dementia while highlighting the ethical implications of their use and the importance of involving all stakeholders to enable a more informed assessment of the benefits and risks and avoid an ageist approach.

The demographic shift cannot be the only justification for using robots with vulnerable populations on account of the “crisis of care” (12) or “care wave” (Horizon Europe project: BB-Future. GA ID: 101093849), which will primarily affect Western societies (13). The demographic transition is progressing more rapidly in South America and Africa than in Europe or areas such as Japan and North America (14). The ethical implications for vulnerable populations are addressed from the engineering and psychological disciplines with the sole aim or justification of improving wellbeing. However, personal, social and cultural consequences must also be reconsidered and analyzed from a broad and interdisciplinary perspective, while the individual’s wishes and decisions must also be taken into account. Despite the demographic transition and the dramatic figures – an estimated 46.8 million people worldwide currently live with dementia, and this figure is projected to double every 20 years to reach 131.5 million by 2050 (15) – the use of robots is not justified unconditionally and requires greater reflection on its ethical implications.

The ethical debate has identified ethical issues associated with using robotics and IA with people with dementia. The need for an ethical approach starting from the technology’s ideation phase has been highlighted. Engineering students, for example, learn ethical concepts and have an interdisciplinary approach to technology so as to raise awareness of ‘embedded ethics’, i.e. the integration of ethics into the whole process from design and development to deployment (16). This is related to the involvement of all stakeholders in the robots’ co-design process. However, in the case of people with dementia this participation is seen as an impediment because of their cognitive problems (17). Fortunately, research is already being conducted to find an inclusive mechanism that enables older adults technological illiterate or with cognitive problems to use new technologies and engage in participative processes by means of a buddy or facilitator (18). Despite these improvements in technology with a human-centric approach, using technological tools such as robots with vulnerable populations is raising doubts and dilemmas. Concerns are appearing regarding the capacity for robots to understand human pain and human needs when this understanding is already difficult for doctors (19).

Among older adults suffering from dementia, individual use of robots has been highlighted as a cognitive tool to act preventively, alleviate loneliness, and improve quality of life (4). Robots have been particularly beneficial in the socio-emotional sphere (20, 21) with significant socio-affective features (22) and the potential to improve engagement (23). While robots are recognized as valuable tools for people with dementia, further comparative studies are warranted (7, 24). Projects like MARIO, part of the European Horizon 2020 program, have illustrated how companion robots can mitigate loneliness and social isolation among older people with dementia (25), while a protocol for the use of PARO has been introduced for older adults with dementia (26). All current high-impact research must obtain approval for implementation from ethical committees to ensure adherence to ethical requirements and data protection standards. However, follow-up studies on the ethical consequences of these studies have not been carried out post-project and deeper analyses of the ethical consequences in the long run need to be addressed.

Other areas of debate relate to the need for the following aspects: transparency, which means understanding the process used by IA tools and communicating well; trust, in the sense that a reliable relationship with the healthcare professional makes the AI tool more acceptable; accountability, with users able to discuss their use; confidentiality, which is problematic because the integration of the healthcare system makes this difficult; autonomy, to avoid paternalistic attitudes and preserve human dignity; and informed consent. Another ethical issue relates to algorithmic bias, especially with regard to gender and race, where, for example, errors in diagnosis have been made and ageist attitudes have been perceived. Finally, fairness is not guaranteed either because economic difficulties mean that access to this technology is not assured (16). Moreover, there are two sides to robotics: although the existence of cheap robots may make them accessible to the population as a whole, those with more financial resources will be able to choose between technocare and human care whereas those with fewer resources will not.

This paper is a review of high-impact articles, mostly in the Web of Sciences and IEEE Xplore databases, that address the use of social robots and companion robots with older people with dementia: in other words, with the vulnerable population. Most papers analyzed are from an engineering or psychological perspective. The search focused on how their ethical implications, dilemmas or challenges have been addressed, what aspects have been highlighted in the research conducted so far, what their limitations are, and what aspects need to be questioned.

In later sections, I outline the characteristics and implications of using social and companion robots with people living with dementia. First, I identify the ethical implications in three interconnected approaches to human-robot interaction (HRI) with older adults with dementia, i.e., the technical, the psychological, and the social. Second, I address the fundamental ethical concepts, issues and problems that have been discussed and those that may arise when social robots and companion robots are used in research with older adults with dementia and also analyze the implications for the various social levels and stakeholders involved in care (older adults, formal caregivers and informal caregivers). Third, I identify research gaps associated with HRI with older adults with dementia and the possibilities for future research. Fourth, I discuss the ethical challenges in implementing robots in social interventions with older adults. Finally, I present the main conclusions of this review.

## 2 Ethical implications in using robots among older adults living with dementia

### 2.1 The ethics of human-robot interaction among older adults: psychological, technical, and sociocultural approaches

During the 1980s and 1990s, robots were mostly a figment of the collective imagination rather than something found in real life. Films commonly depicted robots as agents of the destruction of civilization, while also using the figure of the robot to question the essence, nature, and identity of humankind. By the end of the 20^th^ century, robots were increasingly depicted as humanized entities, grappling with existential questions and striving for autonomy, as depicted in classic films such as *Blade Runner*, *AI*, and *The Matrix.*

Furthermore, the proliferation of robots from industrial services to social functions and companionship has spurred the production of science fiction literature (27–32), with each realm mutually influencing the others. This literature often explores the connections between robotics and ethics, delving into the essence of humanity. For instance, in 2003, Carnegie-Mellon University inaugurated the Robot Hall of Fame, inducting four robots—real or fictional—every two years, an example that underscores the enduring fascination with these objects.

Much of the research on HRI has been conducted from a psychological perspective (20, 33, 34), emphasizing emotional bonds and attachment, particularly evident in interactions with pet robots among older adults or children (35–37). Psychological perspectives regard robotics as therapeutic tools, aiming to enhance cognitive abilities, to engage with people with dementia, and improve quality of life for both patients and caregivers (23, 25, 26, 38, 39). Robots have proven beneficial in the socio-emotional realm, contributing to overall wellbeing (20, 21, 40). Consequently, robots have been viewed positively and are increasingly used to enhance abilities and interactions between individuals with dementia (7, 22). The ethical approach has been based on a substantive rationality following Weberian concepts in which the final aim is to improve the well-being of older adults with dementia by enabling them to communicate with someone or something.

Turning to technical approaches, the field of engineering tends to have machine-centric perspectives on HRI, prioritizing machine viewpoints and focusing on health-related improvements, albeit with less consideration for human outcomes (22). Breazeal (3) characterized social robots from a machine-oriented perspective, emphasizing their social participatory nature and internal motivations: ”Sociable robots are socially participative ‘creatures’ with their own internal goals and motivations. They pro-actively engage people in a social manner not only to benefit the person (e.g., to help perform a task, to facilitate interaction with the robot, etc.), but also to benefit itself (e.g., to promote its survival, to improve its own performance, to learn from the human, etc.)” (p. 169).

Ontological considerations are also pertinent, with robots—particularly anthropomorphic or zoomorphic ones—eliciting expectations regarding their behavior (41, 42). Because of robots’ lifelike features, people—and even animals—expect them to perform value-based or instrumental “social actions” (43) when they interact with us. However, this machine-centric or robotic-centric approach often overlooks clear ethical questions regarding the effects of robotics on human beings, especially vulnerable populations. There is a pressing need for research on how robot implementation can enhance the wellbeing of older adults and their formal and informal caregivers. Technology development is increasingly geared toward addressing the care needs and cognitive improvement of older adults suffering from dementia, epitomized by initiatives such as the CLOHTILDE ERC project (GA ID: 741930) (see: https://clothilde.iri.upc.edu/).

Turning to a sociocultural perspective, in anthropology and sociology the focus shifts toward the individual as embedded in the group, with ethical consequences of the use of robotics with vulnerable populations taking precedence. Robots’ social embeddedness and lifelike traits are analyzed from a sociocultural point of view. Robots prompt contemplation of what it means to be human and the relationships humans have with other living and non-living entities (44–46). This perspective is crucial because—in an expression of ageism—older adults are often viewed as a homogenous category rather than as persons who inhabit a range of cultures, possess different thoughts and beliefs (47), hold preferences, and have the capacity to decide for themselves what type of care they prefer from the options available. This ethical dimension involves ensuring or reinforcing anti-ageist practices.

Furthermore, the exploration of how humans engage with these new virtual beings outside laboratory settings invites an ontological and posthumanist examination of what it means to have relationships with “other-than-human” entities and to extend “sociality beyond the human” (46, 48, 49): What characteristics do we attribute to these virtual beings, and what sort of animisms and ontologies do they inspire? Where is the line between reality and imagination? Analyzing the social embeddedness of robots from a sociocultural perspective allows us to challenge human centrality and superiority. Robots contribute to overcoming anthropocentrism by challenging the dominion of humans over machines and nature, fostering a more egalitarian positioning within the ecosystem, and promoting new attributions based on techno-animism and a posthumanistic approach (44–46).

Finally, little has been said about HRI from an anthropological, sociological or social work perspective in terms of changes in social relationships such as those within families, kinship networks, communities or peer groups. Recent research underscores these gaps in our understanding of cultural differences and introduces this dimension (50, 51). of cultural differences and introduces this dimension. Fundamental ethical concepts, issues, and problems when using robots with older adults with dementia.

Using social and companion robots in the care of older adults with dementia raises several important issues that have been explored in the social sciences and humanities, particularly from phenomenological and anthropological viewpoints. From a social standpoint, studies of dementia often revolve around concepts of personhood and personal identity, questioning whether individuals with dementia continue to be the same persons they were before being affected by the illness, or even if they continue to be persons at all (52). As Stephen Ames (53) notes, understanding “what happens to the person with dementia” depends “on how the person without dementia is understood.” Indeed, definitions of “person” and “personal identity” do not derive directly from empirical reality—and in that sense, they are not “natural”—but rather emerge from historically contingent values and philosophical positions, including brain-based elaborations that were developed in European thought in the late seventeenth century. These perspectives, which present themselves as “scientific,” have come to be defining traits of modernity (54). They emphasize the continuity of memory and self-awareness as criteria of personhood and personal identity (disregarding other criteria, such as embodiment, culture, and intersubjectivity). From this biomedical perspective, dementia is treated as a “death in life or life in death” and the loss of human qualities (55). It is understood as a pathology rather than as another way of living. Research from an anthropological ontological perspective (56) has pointed out that people with dementia are often stigmatized even before the manifestation of severe dementia symptoms, impacting their family and social relationships. Dementia necessitates a readaptation to social life and social relationships. In this sense, both robots and dementia put a mirror before us, leading us to challenge our notions of personhood, humanity, and even life itself.

As Steven Sabat and Alison Warren (57) point out, the emphasis on “memory loss” in describing dementia “connotes an inability to form new memories and participate in meaningful social interactions” (p. 1819), contributing to a diminished sense of self and personhood. As Tom Kitwood and Kathleen Bredin (58) have long argued, “The key psychological task in dementia care is that of keeping the sufferer’s personhood in being,” and this requires seeing personhood in social rather than individual terms. C. Hughes (59), writing on questions of personal identity, personhood and selfhood, states that we aim for “memory to encompass a broader view which emphasizes instead the ability of people to continue to construct their life-worlds through their persisting meaningful relationships” (p. 283). Personhood or a meaningful sense of being, when cognitive capacities are being affected by dementia, can be perceived by the relationship with other beings or objects that can become meaningful or pleasant to us. On a practical level, robots can help older adults participate in social life and in this sense help them, paradoxically, to be a person. At the same time, robots can facilitate interconnection with informal and formal caregivers, thereby becoming a nexus for or creating or improving relationships. This social dimension of robots, which has been explored less, can be positive. However, it can also raise new ethical dilemmas about their use with individuals who cannot provide clear, informed consent or this may be relegated to tutors or other healthcare professionals.

There is a shared optimism regarding the potential of technology to mitigate the limitations caused by the disease, alleviate isolation, and assist in performing the activities of daily living, thereby aiding informal caregivers in managing the care burden. The optimism and hope for positive outcomes are also evident in educational initiatives associated with innovative practices before their implementation. Concerning dementia, the concept of “gerotechnological optimism” is intertwined with values and aspirations but can be tinged with fantasies or wishful thinking. While technology can ameliorate and even help prevent decline and fragility in individuals with dementia (8), it can also be viewed as an illusion, a phenomenon termed “cruel optimism” (9). At the same time, technological optimism is balanced by a techno-pessimistic view and resistance when technology is imposed in caring professions (8, 60, 61).

Despite advances in research, ethical issues persist, necessitating a careful examination of the fundamentally relational processes of HRI and ethical considerations, including social relationships and positionality (62), as well as the “fragility” inherent in the interactions and communication of persons with dementia (63), and the ethnographer’s involvement (or lack thereof) in their interlocutors’ experience (64). Further issues implied in the use of robots concern individuals’ right to decide whether to use the technology, the dynamics of negotiation with end-users, the imperative not to pressure them, the possible reinforcement of ageist attitudes, and unequal access due to illiteracy and unaffordability.

**Table 1** describes some ethical concerns that must be addressed prior to using social robots and companion robots’ for people with dementia, either for empirical research or social interventions.

**Table 1:** Ethical concerns in the use of social robots and companion robots for older adults with dementia.

QUESTIONS TO BE ADRESSED	ETHICAL CONCERNS
1. What are the benefits of social and companion robots for older adults with dementia and for formal and informal carers?	Increase in dependence
	Increase in care work
	Stress and/or rejection of robots
2. How can robots help to address the social and care needs of older adults with dementia in different sociocultural and economic contexts?	Unaffordability of robots
	Rejection of technocare
3. How will older adults with dementia have agency in the decision to use social and companion robots?	Older adults' right to decide
	Advance directives
4. What is the added value of the introduction of social robots and companion robots in nursing homes and long-term care environments?	Lack of privacy
	Older adults' loss of control/external imposition
5. How can this technology ameliorate older adults' loneliness and improve their wellbeing?	Lack of control of their own data
6. What are the cultural and social drivers to be robots' accepted for being incorporated into the social lives of older adults?	Stereotypes about age, culture, class, and gender
7. What kinds of relationships do older adults and other stakeholders develop with these "other-than-humans'?	Difficulties in distinguishing reality from imagination
	Disappointment or even frunstration
8. What are the social roles attributed to social and companion robots, if any, and the systemic implications in different kinship systems?	Difficulties in choosing from robots than human caregivers or having the possibility to complement one with the other
9. What sort of animism and ontologies are developed surrounding social and companion robots?	Difficulties in distinguishing reality from imagination
	Disappointment

These issues should be considered from the perspective of patient-centered care when conducting research and healthcare interventions with robots so as to avoid ageist practices. The use of robotics and AI needs to be legally regulated to ensure ethical compliance, mitigate risks, and safeguard the rights of all stakeholders.

### 2.2 Ethics in artificial intelligence and robotics at the macrolevel

Institutions have the responsibility to regulate the use of AI and robotics in order to preserve an ethical and beneficial use for citizens and, particularly, to respect the rights of the most vulnerable. The European Union (EU) is at the forefront when it comes to the ethical regulation of AI. Aware of the need for public-private partnership (65), it holds a prominent global position in robotics (66). The EU is also at the leading edge of ethical legislation on trustworthy artificial intelligence (AI), with the first legislation on AI being approved by the European Parliament on 13^th^ March 2024 (see https://digital-strategy.ec.europa.eu/en/policies/regulatory-framework-ai) and amendments to regulations (EC) No 300/2008, (EU) No 167/2013, (EU) No 168/2013, (EU) 2018/858, (EU) 2018/1139 and (EU) 2019/2144, and Directives 2014/90/EU, (EU) 2016/797 and (EU) 2020/1828 (Artificial Intelligence Regulation) (see https://www.europarl.europa.eu/doceo/document/TA-9-2024-0138_EN.pdf). Earlier, a white paper on artificial intelligence (67) (see https://digital-strategy.ec.europa.eu/en/library/ethics-guidelines-trustworthy-ai) encompassed robotics and other related technologies. Significant strides have been made in terms of data use and protection, notably with the introduction of the General Data Protection Regulation in 2018 by the EC. Despite these advances, implementing ethics in new technologies is challenging due to different legal structures, technological capacities, and production interests (68).

Efforts are underway to address these challenges through the development of the AI Act, which adopts a risk-based approach to ethical issues from the technology’s initial development phase, emphasizing high levels of robustness, security and accuracy (https://digital-strategy.ec.europa.eu/en/policies/regulatory-framework-ai). Unacceptable risks, such as cognitive manipulation of individuals—especially from vulnerable groups—and the use of personal characteristics for profiling, have been identified. Recognizing the importance of ensuring safety and liability implications in AI, the EP and EC advocate for human oversight and coordinated European commitment and legislation, rooted in a human-centered, ethical, and sustainable approach to AI implementation, robotics, and related technologies (66, 67) aimed at “ensuring AI technologies work for people” (p. 2).

The EU is proactively proposing legislation to regulate both the potential risks and opportunities that robotics and AI imply, including job creation and progress toward the sustainable goals of the European Green Deal (66). For instance, there is a need to explore the potential mental health risks associated with engaging with humanoid robots (67). However, regulations often lag behind the development and deployment of technology (68, 69), necessitating ongoing efforts to ensure safety and trust (70). Addressing ethical concerns during the robot design process (71) through co-ideation and co-validation phases can enhance the acceptability of the final product.

### 2.3 The ethics of care in relation to the use of robots with older adults with dementia

As the use of robots is scaled up to organizational and societal levels, ethical considerations become even more crucial. For example, we must understand how the introduction of robots can affect existing care workers, how affordability may affect access to robots, and how the presence of robots may influence access to and choice of care (72).

According to Alasdair MacIntyre (73), the ethics of care involves interdependence, as people require support from each other at various stages of life. In alignment with this view, Judith Butler (74) describes vulnerability as a “proper condition” of the human being, framing it as a bodily ontology and a “relational social ontology” (75) that operates both at the individual level and as an epistemic framework. This critical perspective sheds light on the violence perpetuated by institutions and underscores how vulnerability is experienced individually yet distributed unequally according to social factors. By acknowledging each other’s vulnerability, the ethical dimension of the concept is also developed. Vulnerability is understood as universal and intrinsic to human existence (76, 77), but it is socially produced and therefore has to be addressed collectively.

Butler’s framework on vulnerability, particularly from a gender perspective, can be structurally adapted to provide a lens through which to illuminate older adults’ specific vulnerabilities, particularly in the face of social actions, new generational dynamics, and technological advancements. From a biomedical perspective, fragility is often linked to bodily health conditions, which in turn influence social vulnerability. In this sense, vulnerability is both biological and social. This underscores, from a social perspective, that human bodies are inherently relational and dependent on each other, rather than autonomous.

Because care is an intrinsic need for all human beings, the provision of care becomes a human right, too. In *Caring Democracy*, Joan Tronto (78) claims that care is a public concern. Both Tronto (78) and Carol Gilligan (79) highlight that there is no justice in democracy without care. The ethics of care necessitates flexibility and adaptability in different contexts, prompting consideration of whether and how to introduce personal robots. Equity and the ethics of care are particularly relevant for low-income countries, where care is crucial to economic development and work opportunities for women.

Previous research has identified several ethical implications: the loss of privacy and safety if robots malfunction, an increase in workload for caregivers tasked with overseeing robot functions, an increase in long-term care costs, and the possible replacement of human care (10, 11). There is growing apprehension that the use of robots could lead to a decline in human interaction for older adults and a consequent increase in dependency (34, 80–82).

Several studies have thoroughly examined these ethical implications. Allaban, Wang, and Padir (10) synthesize these concerns in six general ethical issues: 1) reduced human contact, 2) loss of control, 3) loss of privacy, 4) restriction of liberty, 5) deception and infantilization, and 6) accountability if something goes wrong (p. 11). In the case of older adults with dementia, to implement a Dementia Centered Care techniques, so as to observe whether the use of robots is something pleasant and positive for them or not is an ethical approach to it. There is apprehension about the potential infantilization of older adults, particularly when pet robots are used for older adults with dementia, with men seemingly encountering more difficulties than women who are accustomed to caregiving activities (83). Robots can also be inserted in a group rather than with individuals as a tool to help generate interaction between older adults and their formal and informal caregivers. Not all end-users are necessarily pleased with the use of social or companion robots. Their reactions should therefore be taken into the account when deciding whether robots should or should not be used in each case. A non-ageist approach means not making assumptions and not imposing this technology even on older adults who are living with dementia.

The ethical implications of using social and companion robots among older adults are greater than in the case of using robots in industrial settings (31, 84). The acceptance of robots is another ethical controversy underlined by scholars (31, 85, 86). This issue warrants further analysis to determine under which conditions robots can be ethically employed. It prompts us to ponder whether their use is legitimate given the imperative to address “the crisis of care,” especially for vulnerable populations such as older adults with dementia. There is an ethical concern over the threat to human dignity when technocare is used with frail and vulnerable older adults with dementia, especially when there is no clear informed consent or preference regarding its use.

Both barriers and facilitators have been identified. Privacy concerns appear to be less prominent since no private data is utilized, especially in the case of companion robots. However, other ethical issues arise: for instance, a user’s potential inability to distinguish between reality and imagination or between a machine and an animal, which can cause disappointment when the machine has fewer functions than expected. The replacement of human caregivers by robots is another complex issue that necessitates examination to determine the conditions under which it can be accepted. Additionally, social justice considerations, such as equitable access to this technology, warrant highlighting (72).

The use of social and companion robots seems more questionable than that of assistive robots, which fulfill specific care needs or provide physical help. Not surprisingly, there is worldwide cultural variation in how people accept the use of technology for activities traditionally associated with love and care (86). Research shows it to be controversial in Europe (87).

Robots highlight ethical questions about our care values, the allocation of resources, and the pursuit of collective wellbeing. In this sense, they have implications for substantial rationality or formal rationality following Weberian terms (43). The final aim and the values surrounding it are important elements to take into account to ensure an ethical approach. Likewise, Aristotelian virtues, which are placed in the social and community life and individual framework of human beings (73), are discovered in the inherent goodness of each being. The telos, or final aim, serves as the yardstick for ethical deliberations concerning robots and object relations, intertwined with human rationality. From a psychological and biomedical standpoint, implementing robots for older adults with dementia may prioritize cognitive enhancement as the end goal, potentially overlooking other consequential factors, ontological considerations, or even the patient’s wishes.

From a social perspective, significant ethical dilemmas emerge regarding the consequences of integrating robots into care practices, following Weberian substantial rationality. This entails considering a blend of values concerning the nature of care and how it is provided. In this sense, stances toward technology are ambivalent: it is both a sign of progress in society and a harbinger of a dystopian future. An initial epistemological question arises regarding the implied obligation to use disruptive technology simply because it has been developed. This underscores the necessity of an ethical approach from the inception of the research process, weighing the appropriateness of adopting such technology for development while ensuring alignment with people’s needs (88). Furthermore, ethical issues arise concerning the implementation of robotic technology as substitutes for human caregivers of vulnerable patients, particularly in the absence of clear consent by the end-user. The attribution of agency to these virtual beings by older adults with dementia, for whom the boundary between reality and imagination is blurred, can generate confusion and stress. This aspect requires careful consideration in deciding how to use this technology, among which end-users, and under which conditions.

### 2.4 The acceptance of social and companion robots among older adults

The acceptance of robots presents an ethical issue that requires careful consideration, particularly in the context of older adults with dementia. Research has indicated a clear lack of acceptance of social and companion robots among older adults without care needs. This reluctance stems from concern about the robots’ lack of authenticity, fears of losing independence and being replaced by machines, or the inability to maintain control over the situation (89–91). Interestingly, even older adults with higher education levels who do not have care needs exhibit similar hesitancy toward robot acceptance (92, 93). However, robots seem to be more accepted when they have a specific purpose and task. Factors such as their functionality and appearance have been identified as crucial contributors to their acceptance among older adults (90). Nonetheless, further research on the social implications of robots and their acceptance is still needed (51, 86, 91).

The robots’ appearance, as noted by Savery (94) is significant in their acceptance, but perhaps even more crucial is the range of care needs they can address, which appears to strongly influence acceptance among older adults (34, 95, 96). Additionally, technological literacy plays a vital role in improving understanding of and trust in robots (97, 98).

Among older adults, there is a preference for social robots that offer services rather than merely providing companionship. Consequently, humanoid or anthropomorphic forms are more likely to be rejected, especially if they are programmed to simulate a particular person, because of their lack of authenticity (89, 90). Some recent studies also identify gender differences, with women showing more interest in pet-type robots, while men tended to prefer humanoid forms (99).

The acceptance of social and companion robots among older adults with dementia has not been sufficiently addressed in the existing literature. Most studies draw conclusions as if acceptance were taken for granted simply because these robots are seen as disruptive technology (100). Acceptance is often viewed through the lens of the tool’s adequacy in addressing cognitive impairment and social isolation from a psychological perspective (6, 25, 38), the perceived benefits in enhancing quality of life (5, 23, 34, 72), or the recognition of the robots’ attributes (39). Although the attitude of end-users when interacting with robots is considered, the research tends to take for granted that users will accept the robots and focus instead on personal preferences in how to use them.

It must be stressed that obtaining informed consent can be challenging in the case of older adults with dementia, requiring a guardian to act on their behalf. However, advance directives could potentially address this issue. Currently, non-verbal indicators of users’ attitudes toward the robots are used to judge whether consent has been given. This awareness can extend to a broader understanding of the ethical dilemmas surrounding the attitudes of patients who are displeased with the use of robots. Some patients may express indifference or lack of understanding toward robots, perceiving them as meaningless or failing to see their purpose. While this may not necessarily indicate clear disapproval or rejection, it casts doubt on their acceptance of the technology.

### 2.5 Robots’ relationship with informal and formal caregivers

The near-future scenario of the “crisis of care” (101) or “care wave” is characterized by an escalation in the care burden, exerting significant pressure on informal caregivers, particularly women, and increasingly younger people who must take on the role of informal caregivers of their parents and/or grandparents. The crisis of care has multifaceted consequences, impacting the labor market, quality of life, and the health and wellbeing of both formal and informal caregivers. It necessitates a substantial increase in care service provision from both the public and private sectors to address the growing demand for care, placing immense pressure on the welfare state (102, 103). Assistive robots with rehabilitation functions or cognitive tools are seen as a way to relieve the care burden on informal caregivers who care for older adults with dementia. Social robots, on the other hand, have been used for remote control and entertainment (104), while the function of companion robots as pets to provide entertainment and play or give caregivers a rest, lies in the emotive and caring dimension itself.

Some caregivers are optimistic about new technology (105),viewing companion robots as a tool to alleviate caregiving responsibilities, increase caregivers’ usefulness (106), and potentially increase the happiness of end-users (39), while also easing the burden of care work. However, it is important to note that in the case of pet-type robots, caregivers are cognizant that some end-users with dementia may reject the robot, experience stress, or simply not take to it (7). This reluctance may sometimes be attributed to individuals not liking animals, rather than the robot itself (72). Regardless, the use of robots for caregiving activities requires adequate training (106). Furthermore, it is crucial to consider the interests of caregivers, particularly informal caregivers, in the design and functionality of robots (107), as they are integral participants in the caregiving process and the care relationship.

The use of robots in nursing homes introduces changes in work organization and creates new tasks, posing certain barriers (108). The high cost of robots means that discussions should take place about robot-sharing (72), which in turn necessitates conversations about how to prevent infections as robots move from patient to patient (83). There are both advantages and disadvantages for formal caregivers, with benefits such as entertainment and cognitive improvement countered by the need for constant supervision and technical assistance, leading to additional work for already busy care workers and therapists (72, 105). (In this sense companion robots such as PARO could be a good option because of their ease of use). Moreover, the costs of technology present a significant barrier to implementation, as robots may not be affordable for all nursing homes, although lower-cost options are available on the market (72).

Despite these barriers and the burnout experienced by many residential staff and care workers, there is a tendency for care workers and healthcare professionals to be more receptive to collective staff activities than individual ones. In this sense, they may be willing to share their experiences with colleagues and, in doing so, innovate with new technological approaches. They also show a willingness to engage in partnerships with professionals outside their institutions (36), facilitating the exchange of views and practices and enhancing their professional relevance as a collective (see https://www.socatel.eu/wp-content/uploads/2021/06/D5.2.pdf).

In addition to the aforementioned considerations, concerns regarding safety in the use of robots have been raised, as their use may pose risks to users (39, 109, 110), and there is potential for problem behaviors. For example, some robots may make it possible for end-users to access gambling platforms. These new ethical dilemmas surrounding the use of robots by patients present challenges for both formal and informal caregivers (107). Despite some negative aspects identified by healthcare workers, such as the infantilization of patients (especially those without cognitive impairments), robots are also seen as a tool for supporting everyday care. Considering the specific factors of each nursing home context is increasingly important to face organizational needs, limitations and drivers. This approach is essential for the social acceptance of this emerging disruptive technology. Involving formal caregivers from the beginning of the co-design phase is crucial (71, 72, 110). As familiarity with the robots grows, so does their acceptance among formal caregivers.

The use of robots for care raises concerns about the potential reduction of human contact, prompting caregivers to reconsider the nature of their roles and how they fulfill them (111). Furthermore, this type of technical care can be perceived as a replacement for human caregivers, potentially leading to the dehumanization of care (72) and the loss of care jobs. The use of robots could be seen as a way of “entertaining” patients, without requiring constant support from caregivers and necessitating only minor supervision.

From an ontological perspective, employing robots with vulnerable populations also challenges the fundamental meaning of care and raises questions about what constitutes optimal care. Can we equate “human care” and “technocare”? Can robots be used effectively for caring for older adults with dementia? Can they substitute human caregivers? Should end-users have a choice in the matter? These are complex issues that society and individuals must confront, and responses may vary across different social contexts and cultural perspectives.

## 3 Research gaps and future research on the ethics of using social robots and companion robots among older adults with dementia

Studies in gerotechnology and science and technology have underlined the need for extensive research in the intersection of aging studies and technology across various disciplines in the social and health sciences (112). Despite positive outcomes in research using social and companion robots (34, 72, 95), some voices have expressed concerns regarding the excessive orientation toward technological solutions in care for older adults and argue that ethical dilemmas have not been solved, for instance in the use of pet-type robots (72).

Interdisciplinary cross-cultural research is essential to maximize the benefits and reduce the risks (113). Critical perspectives argue against the use of robots to care for people with dementia, drawing parallels with the rejection of using robots to care for children, regardless of the reasons (87). However, examining the issue from a cross-cultural standpoint reveals variations in attitudes and practices. For instance, low-cost robots *have* been used to care for children, for example in Korean preschools. Likewise, in Japan there seems to be less resistance to the deployment of social robots (113, 114). Large-scale comparative studies are essential, especially ones that test robots in real-world settings, rather than laboratory environments (50, 115). Sociocultural backgrounds also seem to play a role in the acceptance of robots, although research on cross-cultural aspects and HRI remains limited (50, 51, 116). Existing comparative analyses have primarily focused on reactions to design features, particularly among students and children, rather than adopting a gerotechnological approach that emphasizes the role of such technology in welfare or care.

The current focus of research remains predominantly centered on cognitive therapeutic interventions (117), such as the implementation of psychological protocols (26), rather than adopting a holistic and social approach that considers social and family relationships, as well as kinship implications in the use of social and companion robots. Further investigation into the social and cultural implications of using these robots is still necessary, with impact extending beyond academia to the broader society. It is imperative to consider the effect of social robots on older adults with dementia in various global contexts, while also assessing environmental trade-offs in terms of energy consumption and waste management (118), as well as cloud connectivity, where applicable. Additionally, there is a need to address country and regional differences and inequalities in access.

Most research on the ethics of robot deployment has predominantly focused on the service provided, neglecting to explore the social functionalities of robots and the dynamics of human interaction and relationships with them. Often, robots are viewed solely in their capacity as “assistive technology” or “welfare technology” (this last term used particularly in Nordic countries) (50, 119), disregarding their potential social and community-transformative roles in fostering kinship, friendships, and community relationships. The anthropomorphism of robots and the ontological phenomenology and animism attributed to these new virtual beings raise ethical concerns (120), as such attributions can make humans act differently. In particular, older adults with dementia may develop expectations and attachments that may result in disappointment if continuity is not ensured. Additionally, it is essential to consider the infantilization effect and the gender perspective, examining how the utilization of robots and their acceptance vary among older women and older men affected by dementia (83). Furthermore, there persists an ageist attitude toward older adults with dementia (which also applies to older adults in general), leading them to be treated as a homogeneous category without considering differences in gender, culture, age and educational background from an intersectional perspective.

Studies based on short periods of exposure to robots highlight the need to investigate prolonged use of robots among older adults (7, 121). Such research should aim to better understand the outcomes following these initial encounters with robots: the routinization—in the Weberian sense—of living with robots; in other words, becoming accustomed to them. Additionally, a new question arises regarding the possibility of expanding access to this technology beyond technologically advanced societies in the West and Asia, including the testing and assessment of robotics in Africa and Latin America. The global population should not be hindered from deploying robots and providing feedback, allowing researchers to adopt a more egalitarian approach and avoid biases in robot design. Moreover, the use of social robots should be a matter of choice and not be imposed, directly or indirectly, due to socioeconomic reasons or any other factors.

There is a notable lack of inclusion of older adults—particularly those living with dementia—in the design, development and implementation phases of social and companion robots (50). Utilizing participatory methods and co-creation and co-development techniques is essential to ensure a more effective deployment that is age-friendly and dementia-friendly, thus making robots a more familiar tool while addressing the needs and wishes of end-users and both formal and informal caregivers. Innovation in co-design necessitates the participation of people with dementia in all phases, including the analysis of data and interpretation of results (122). Including people with dementia in social research with older adults is not common. While it may be desirable to include individuals with dementia as a target population to reflect the variety among older adults’ typologies and conditions, this is often avoided due to ethical complexities. The necessity of including such individuals must be well argued. Meeting all ethical requirements can be challenging for researchers, necessitating careful consideration to avoid substantial complications.

Participatory methods and community involvement serve a dual purpose: addressing the needs and desires of individuals while also enhancing robots’ age-friendliness and dementia-friendliness. Additionally, these approaches facilitate the social inclusion of people with dementia within a community-based care framework. Inclusion also extends to both formal and informal careers, practitioners, and family members, who should participate in all research phases, including discussions about ethics and the promotion of sustainable engagement (72).

## 4 Ethical dilemmas in the implementation and assessment of robots among older adults with dementia

When it comes to implementing services for older adults with dementia, there is an emphasis on offering cognitive-oriented activities that present minimal disruption and cost. Proven effectiveness has been considered a sufficient justification to proceed with implementation, a premise that raises ethical questions and needs further analysis, as I have outlined throughout this article. Setting aside for now the issue of whether robots *should* be implemented in care for older adults living with dementia, I turn to issues that must be resolved if implementation were to proceed.

A distinction has been made between the biomedical approach, which tends to control and isolate the patient, and a more social, community-based approach that adopts a holistic, dementia-friendly view of personhood, based on selfhood and the individuality of each patient. From a biomedical standpoint, individuals with dementia are often viewed as unable to produce research knowledge, and there is a noticeable disparity between pre-diagnosis and post-diagnosis phases. The term “dementia” carries stigma, leading some to prefer the term “memory problems.” However, consistency in terminology is crucial for accuracy in publication, which itself has ethical implications (123). The contrast between biomedical approaches and community-based care is evident. While biomedical diagnosis can inadvertently act as a self-fulfilling prophecy, exacerbating the severity of the disease by shaping the social construction of the illness and influencing family and social relationships, community-based care—which is person centered—emphasizes attention to non-verbal communication of older adults with dementia, uses empathy and inclusion, and fosters autonomy. This approach embraces flexibility or “going with the flow” to adapt to the day-to-day situation (56).

Despite the increasing recognition of ethical concerns surrounding how to protect older adults living with dementia, there remains a disparity in focus between health sciences and social sciences. The importance of involving caregivers in research and incorporating their views is gaining prominence in both medical and social research, particularly considering the emotional implications and bonds formed with individuals living with dementia. This blurs the lines between the roles of researcher and caregiver in this humanistic and participatory approach. The researcher may assume a caregiver-like role due to this ethical involvement (124). Furthermore, the boundary between formal caregivers and researcher is often blurred, because interacting with older adults as a researcher often requires having some degree of care training. This situation raises post-project ethical implications, such as what happens after a short-term intervention with robots in a nursing home, in which people with dementia may have created bonds with these animated virtual beings and the researchers. As described above, research has indicated that older adults prefer social robots that provide services rather than only companionship (89, 90), and women prefer pet-type robots, while men prefer humanoid robots (99). However, before rollout, such generalizations would require testing across different sociocultural contexts, considering diachronic changes in gender values. Limitations in older adults’ technological literacy—and their awareness of stereotypes about it—may produce embarrassment and anxiety when using robots, necessitating open dialogue and the development of user-centered experiences (125), particularly when the target users have dementia.

The implementation of robots would also need to navigate collective fears and uncertainties surrounding disruptive and unfamiliar technology. While it is crucial to consider age and cognitive abilities when designing social and companion robots, it is perhaps even more important to consider the sociocultural context in which they will be used (10). Additionally, multiple barriers exist at the organizational level, as mentioned above, which need to be addressed during implementation (108).

In the context of COVID-19, while other types of robots, such as telepresence robots, saw increased use, serving to facilitate exercise and enhance technology utilization overall—a silver lining in the pandemic (126)—the utilization of social and companion robots for people with dementia decreased. This decline can be attributed to the overwhelming situation faced by staff and the fact that robots are best suited for use in the early stages of dementia (127). The potential use of these robots in family settings has yet to be fully investigated. There may be reluctance stemming from the fact that so far, the use of robots is in an experimental phase.

One region of the world where robot implementation has begun to take place is the Nordic countries, through various municipal programs that align with the goal of welfare technology development (110, 128). Political discourse supporting welfare technology has encouraged care workers to embrace care robots that align with their professional values and that are deemed useful, for instance in dispensing medication (110), as a means to cope with increasing care needs.

As robots transition from laboratory settings to societal implementation, it is crucial to consider the wishes of older adults with dementia and to discuss the ethics of robotics in their care (72). One tool may be the use of advance directives in which an older person’s wishes are recorded. Moreover, older adults with dementia should be included in the design and deployment phases of interventions, which can be aided by trained facilitators (18).

## 5 Discussion and conclusions

Despite considerable advancements and pioneering research on the utilization of social and companion robots for older adults with dementia (5, 6, 40), there is a pressing need for comprehensive analysis from a social sciences perspective regarding the ethical implications and repercussions of HRI in this context. This analysis should prioritize ethical awareness, while assessing the appropriateness of employing such technology to confront the impending “crisis of care” and the loneliness and isolation experienced by older adults with dementia.

From a gerotechnology perspective, ethical concerns persist regarding the use of robots with vulnerable populations. One key issue is the right of each individual to decide whether to use this technology, with decisions being negotiated rather than imposed. We must ensure that the use of robotics in care, particularly for vulnerable populations, such as older adults with dementia, is accepted and potentially included in advance directives. Additionally, there is the risk of reinforcing ageist attitudes by treating older adults as a homogeneous group. Barriers such as illiteracy and unaffordability and difficulties in distinguishing between reality and imagination can question the wisdom of incorporating such technology. We need to decide whether to use technocare tools and determine their role and importance in fulfilling care needs. Issues such as the replacement of human caregivers and the impact on employment opportunities also need to be considered.

There is also a need for greater involvement of formal and informal caregivers, older adults, and older adults with dementia from the inception of the research process, including the co-design phase. This approach aims to address real needs and avoid possible risks in the use of robots for older adults with dementia, ensuring their utility. It is important to anticipate all possible ethical implications from the initial design of the research and to follow up on any ethical concerns that arise during and after the research project. For example, removing robots after a successful but limited period without providing alternatives can pose significant ethical challenges. Also, older adults should be incorporated from the technology co-ideation and co-design phase in order to introduce a human-centric approach.

Ethics must be at the core of research and social interventions, addressed from the very beginning of the design of the research and followed throughout the entire process. Ethical debate should be open and should include the participation of all stakeholders involved in care. Currently, there are no specific guidelines with a practical focus on ethical research that promote a personhood-holistic approach and involve citizens. This approach should aim to raise awareness of ageist and stereotypical misconceptions that lead to the exclusion of people with dementia from research due to cognitive challenges (120). Empirical research is needed to better test and understand the use of social and companion robots with this population. A human-centric approach to technology that involves the participation of all stakeholders throughout the process—from co-ideation, co-design and co-development through to deployment—can ensure that an ethical perspective is applied to a more respectful, age-friendly and dementia-friendly approach to robotics. Dementia-centered care must also be included to ensure personalized enjoyment and acceptance of robotics while testing and/or using social and companion robots with older people with dementia.

Numerous experiments have used social and companion robots with people living with dementia in Western societies (6, 7, 26, 72) from therapeutic and psychological perspectives. However, there is a lack of research on the social implications, including potential biases and stereotypes related to gender, culture, age, and education. Major comparative studies are needed to consider social and cultural diversity in research involving robots. Additionally, there should be more international exchange of knowledge and experiences to improve implementation and share best practices.
